# A case of juvenile myoclonic epilepsy in which a disease‐specific question led to the diagnosis

**DOI:** 10.1002/jgf2.496

**Published:** 2021-09-23

**Authors:** Hiroki Maita, Tadashi Kobayashi, Takashi Akimoto, Hiroshi Osawa, Hiroyuki Kato

**Affiliations:** ^1^ Development of Community Healthcare Hirosaki University Graduate School of Medicine Aomori Japan; ^2^ Department of General Medicine Hirosaki University School of Medicine & Hospital Aomori Japan

**Keywords:** disoriented state, myoclonic jerk, postictal symptom, seizure, syncope, twilight state

## Abstract

A 17‐year‐old female patient presented to our hospital with repeated transient loss of consciousness lasting less than 10 min. After regaining consciousness, she experienced no disorientation, confusion, tongue‐biting, or incontinence. Physical findings, blood tests, electrocardiogram, and echocardiogram showed no obvious abnormalities. On being asked whether she had experienced sudden rapid body movements, she answered “yes.” Therefore, we suspected juvenile myoclonic epilepsy (JME) and obtained an electroencephalogram, which showed diffuse bilateral bursts of spike‐and‐wave complexes, confirming the diagnosis. In adolescent patients with transient loss of consciousness, myoclonic jerks should be actively confirmed for the diagnosis of JME.

## 1 INTRODUCTION

Juvenile myoclonic epilepsy (JME) is a common form of generalized epilepsy that occurs in otherwise healthy adolescents and presents with the triad of myoclonic jerks, generalized tonic‐clonic seizures, and absence seizures. The prevalence of JME is estimated to be 0.1 ~ 0.2/1000,^1^ and primary care physicians are likely to encounter this disease. The diagnosis of JME is usually uncomplicated because of its characteristic history. However, transient disturbances of consciousness and generalized tonic‐clonic seizures are the main complaints at initial presentation, and myoclonic jerks are often not reported. The interval between JME onset and an accurate diagnosis ranges from 2 to 8 years.^2^, ^3^ Thus, awareness of the characteristics of JME is important to ensure prompt diagnosis and treatment and avoiding incidents caused by loss of consciousness (LOC).

Generally, when treating patients experiencing transient LOC, differentiating between epilepsy‐induced seizures and syncope is important.^4^ Primary care physicians make diagnoses based on a detailed history, including the duration of LOC, prodromes including presyncope and diaphoresis, physical findings including tongue biting, and the characteristics of postictal symptoms,^4^, ^5^ although differentiation is sometimes difficult. The mean duration of impaired consciousness due to first‐epileptic seizures in children is 12.2 (standard deviation, 26.9) min,^6^ and disorientation and confusion (also known as the twilight state^7^) following seizures are reported to occur in 51–85%^8^ and 94%^5^ of patients, respectively. Most postictal symptoms, including headache, confusion, and paresis, improve within 24 h; however, psychosis may persist for months.^9^ In this report, we describe a case of JME, in which we initially suspected syncope because of the short LOC and absence of postictal symptoms; however, a history of characteristic myoclonic jerks led to the diagnosis of JME.

## 2 CASE

A previously healthy 17‐year‐old female patient with no perinatal or developmental problems presented to our hospital for transient LOC. Twenty‐nine days before her visit, she experienced sudden LOC at the station on her way to school. She recovered consciousness in about 5 min and was able to go to school as usual. She experienced no signs of disorientation, confusion, tongue‐biting, incontinence, or paralysis after LOC. She visited a community clinic later that day, although the cause was not identified. Eight days before visiting our clinic, she experienced brief nausea and LOC for <10 min again at the station. After regaining consciousness, she experienced no postictal symptoms and was able to walk home unaided. She was subsequently referred to our hospital for evaluation.

The patient had been taking ibuprofen once a month for mild headaches and menstrual cramps and had never experienced chest pain, palpitations, or dyspnea. She showed no edema, weight gain, obvious physical or neurological abnormalities, or orthostatic hypotension. Blood tests indicated no abnormalities (Table 1). A plain chest radiograph, electrocardiogram, and echocardiogram showed no abnormalities. When she returned 1 week after the initial visit, we inquired if she had experienced any sudden involuntary body movements to confirm the presence of myoclonic jerks. She stated that she had occasionally experienced sudden movement of her legs while taking a class or operating her cell phone, that the movements appeared at least once a month, and that they had occurred at least four times so far. Based on the patient's age and the history of characteristic myoclonic jerks, we considered JME to be the primary differential diagnosis and obtained an electroencephalogram, which showed diffuse bilateral bursts of spike‐and‐wave complexes, leading to a definitive diagnosis. She consulted an epilepsy specialist, and after taking an initial dose of lamotrigine 25 mg/day, which was later increased to 100 mg/day, the myoclonic jerks disappeared, and no recurrence of LOC was observed for >2 years.

**TABLE 1:** Hematological and biochemical investigations

	29 days after the symptom onset (the date of the patient's first visit to our hospital)
White blood cell count (/μl)	4590
Neutrophil (%)	50.2
Lymphocyte (%)	42.7
Monocyte (%)	4.4
Eosinophil (%)	2.0
Basophil (%)	0.7
Red blood cell count (×104/μl)	427
Total protein (g/dl)	6.9
Albumin (g/dl)	4.3
Blood urea nitrogen (mg/dl)	17
Creatinine (mg/dl)	0.72
Sodium (mmol/L)	139
Potassium (mmol/L)	4.1
Chloride (mmol/L)	106
Calcium (mg/dl)	9.0
Aspartate aminotransferase (U/L)	15
Alanine aminotransferase (U/L)	11
Alkaline phosphatase (U/L)	87
Gamma‐glutamyltransferase (U/L)	7
Lactate dehydrogenase (U/L)	125
Creatine phosphokinase (U/L)	56
C‐reactive protein (mg/dl)	<0.02
Glucose (mg/dl)	117
Thyroid‐stimulating hormone (μIU/ml)	0.79
Free triiodothyronine (pg/ml)	3.21
Free thyroxine (ng/dl)	1.37
Cortisol (μg/dl)	3.97
Antinuclear antibody	Negative

## 3 DISCUSSION

This case report presents two important conclusions. First, in adolescents with transient LOC, especially when seizures are likely, JME should be suspected and the presence of myoclonic jerks should be confirmed. Although the first generalized tonic‐clonic seizure in JME is often preceded by myoclonic jerks for several months to several years,^10^ patients typically visit a physician after their first generalized tonic‐clonic seizure^10^ and usually report only the seizures. Therefore, medical providers should actively suspect JME and specifically inquire about it. Myoclonic jerks reportedly occur most frequently after morning awakening, after naps, and intermediate awakenings during the night.^10^ Although myoclonic jerks do not necessarily indicate certain diseases, when examining a patient with impaired consciousness or suspected epilepsy, the specific question “Have you experienced any sudden involuntary movements and dropped things as a result?” can facilitate the diagnosis of JME. Patients with JME generally respond rapidly and completely to broad‐spectrum antiepileptic drugs, such as valproate or lamotrigine.^10^ Thus, prompt diagnostic planning and early treatment can avoid accidents caused by LOC and improve the daily lives of adolescents. In many cases, valproic acid is the first‐line drug because of its efficacy in treating seizures and its low cost. However, it is teratogenic, and other agents such as lamotrigine or levetiracetam may be recommended in postpubertal women, especially those considering pregnancy.

Second, as syncope and seizures cannot be distinguished purely based on the patient's history, seizures must be considered even if the LOC is transient or presents without subsequent disorientation or confusion. In a report analyzing transient LOC in patients with seizures, the sensitivity, specificity, and negative likelihood ratio of LOC longer than 5 min were 68%, 55%, and 0.6, respectively, indicating that the possibility of a seizure cannot be ruled out even if the LOC is <5 min.^8^ The existence of a disoriented state evaluated by an eyewitness showed a sensitivity, specificity, and negative likelihood ratio of 85%, 83%, and 0.2, respectively, making it difficult to rule out seizures based on this alone. Exclusion of seizures is even harder in cases with a self‐reported disoriented state because the diagnostic accuracy decreases significantly (sensitivity, 51%; specificity, 91%; negative likelihood ratio, 0.5).^8^ To differentiate seizures from syncope, the diagnostic approach should combine multiple findings, rather than being potentially confused by the vagaries of postictal symptoms, especially if the patient self‐reports them.

In conclusion, seizures cannot be ruled out in patients with transient LOC even if the episodes are short or do not involve disorientation or confusion, especially when the information is self‐reported. In adolescent patients, JME should be considered in the differential diagnosis, and primary care physicians should actively and specifically investigate the presence of myoclonic jerks.

## CONFLICT OF INTEREST

The authors have stated explicitly that there are no conflicts of interest in connection with this article.

## INFORMED CONSENT

We obtained written informed consent from the patient to publish this case report.
